# Selection and Characterization of Potential Baker's Yeast from Indigenous Resources of Nepal

**DOI:** 10.1155/2017/1925820

**Published:** 2017-12-13

**Authors:** Tika B. Karki, Parash Mani Timilsina, Archana Yadav, Gyanu Raj Pandey, Yogesh Joshi, Sahansila Bhujel, Rojina Adhikari, Katyayanee Neupane

**Affiliations:** ^1^Department of Biotechnology, Kathmandu University, Dhulikhel, Nepal; ^2^Biotechnological Research and Development Center Pvt., Ltd., Bharatpur, Nepal

## Abstract

The study aims to isolate the yeast strains that could be used effectively as baker's yeast and compare them with the commercial baker's yeast available in the market of Nepal. A total of 10 samples including locally available sources like fruits, Murcha, and a local tree “Dar” were collected from different localities of Bhaktapur, Kavre, and Syangja districts of Nepal, respectively. Following enrichment and fermentation of the samples, 26 yeast strains were isolated using selective medium Wallerstein Laboratory Nutrient Agar. From the differential tests which included morphological and microscopic observation and physiological and biochemical characterization such as nitrate reduction and lactose utilization tests, 8 strains were selected as possible* Saccharomyces* strain. The selected strains were further assessed for their efficient leavening ability by tests such as ethanol tolerance, osmotolerance, invertase test, and stress exclusion test. The three most potent strains ENG, MUR3B, and SUG1 isolated from grape, Murcha, and sugarcane, respectively, were used in the fermentation and baking of dough. These strains also carried a possibility of being used as industrial baker's yeast.

## 1. Introduction

*Saccharomyces cerevisiae*, the microorganism used in baking industry as a leavening agent [1], with the advancements in bread industry, has been continuously improvised for decades [2]. The main yeast strain is commonly reported to be responsible for alcoholic fermentation [3]. This yeast utilizes the hexose sugars especially maltose to produce CO2, ethanol, and variety of secondary metabolites such as esters, aldehydes, and amino acids that contribute to the development of flavor and aroma of the fermented food [4, 5]. The carbon dioxide thus produced is responsible for not only increasing the volume of dough (leavening process) through gas incorporation but also a value addition to the flavor and texture [6]. Besides, the fermentation products like vitamins and amino acids are responsible for the health and nutritional benefits that are obtained from bread [7].

Yeast exists in natural environment like plant tissues, fruits, grains, leaves, dung, soil, and other fermented products [8]. The best source of yeast is considered to be citrus juice [9] and sugarcane juice [10]. Yeast strains present on fruit surfaces are able to ferment variety of sugars to alcohol and can withstand high alcohol concentration.

Although the consumption of baker's yeasts in Nepal is rising every year, the country imports its entire requirement of baker's yeast mostly from China and Europe. Nepal imports the baker's yeast of 10 million US dollar worth in 2013 [11]. Nepal, being a country with wide diversity of flora and fauna, has a high possibility of inhabiting unique strains of baker's yeast which is yet to be explored. Considering the economic importance of yeasts in baking industries of Nepal, the following study was done to isolate potential baker's yeast strains with leavening properties from various local sources. Different physiological properties of yeast such as invertase activity, flocculation, ethanol tolerance, hyperosmotic tolerance, temperature tolerance, and CO_2_ production for a strain to be used as a commercial baker's were also analyzed [12, 13].

*Boehmeria rugulosa, *commonly known as Dar or Githais, an indigenous tree grown in Nepal from 300 to 1700 m, is most common in the higher elevation* Shorea robusta *forests and in* Alnus nepalensis* forests at about 1500 m [14]. The bark is dark brown in color and very rough and deep fissures render it into small, more or less rectangular scales [15]. People in Parroha VDC, Rupandehi, mix a paste or powder of* Boehmeria rugulosa *bark with rice flour to prepare Sel-Roti, typical Nepali ring bread generally used during festivals [16]. Since the members of Saccharomycetales are also present in the bark of certain deciduous trees [17], there is a possibility that the bark of Dar may contain some* Saccharomyces *species.

Murcha is a mixed starter culture which contains mixture of fermenting yeasts, saccharifying molds and acidifying bacteria [18], which is used as an inoculum for making local alcohol (jand) in Himalayan regions of Nepal, India, Bhutan, and Tibet [19, 20]. Murcha is a round cake which is mildly acidic with pH about 5.2 [21]. It is traditionally prepared from wild plants that harbor microorganisms needed for the amylolytic fermentation (Rai and Subba (2003)).

## 2. Materials and Methods

### 2.1. Materials

Ten samples which include 6 fruit samples, that is, sugarcane* (Saccharum officinarum)*, apple* (Malus domestica)*, grapes* (Vitis vinifera)*, jackfruit* (Artocarpus heterophyllus)*, mango* (Mangifera indica),* and papaya* (Carica papaya)*, soil, Murcha, bark of “Dar”* (Boehmeria rugulosa)*, and commercial active dry yeast powder as reference were collected. The samples were aseptically stored at 4°C.

### 2.2. Isolation and Preservation of Yeast Colonies [22, 23]

Enrichment was done to increase the number of native microflora present in the sample by adding 27% (w/v) sucrose in the crushed fruit samples and was allowed to ferment for 3 days. The prepared samples were plated on Wallerstein Laboratory Nutrient Agar medium supplemented with chloramphenicol (0.01%) at pH 5.5 and allowed to grow for 72 hours. Through morphological examination, two distinct yeast colonies, that is, white and green, were selected. The selected colonies were subsequently subcultured on Yeast Malt (YM) Agar medium supplemented with chloramphenicol (0.01%) to obtain pure isolates.

Maltose is the principle carbon source during bread fermentation. Thus, baker's yeast strains are good utilizers of maltose [24]. The presence of maltose as the primary sugar source in YM media and final pH set to 4.6 ± 0.1 (optimum pH for* Saccharomyces *strain) also act as screen as it enhances the growth of baker's yeast.

### 2.3. Microscopic Observation

A single colony of yeast was mixed in a droplet of sterile distilled water on glass slide and smeared until the smear dried off. The smear was then stained using diluted lactophenol cotton blue dye, air-dried, and observed under light microscope at 100x magnification.

### 2.4. Screening

#### 2.4.1. Nitrate Reduction Test [25–27]

A well isolated colony was inoculated in nitrate broth (peptone 10 g, KNO3 10 g in 1000 ml distilled water). It was incubated at 30°C for 48 hours. After incubation, 5 drops of both reactive 1 (*α*-naphthylamine 1 g, distilled water 22 ml, heat solution, filter, and then adding acetic acid 1 ml) and reactive 2 (sulphanilic acid 0.5 g, diluted acetic acid 150 ml) was added in the tube. The appearance of red color was observed after 5–10 minutes.

#### 2.4.2. Lactose Utilization Test [28]

Yeast cells were grown at 30°C for 3 days into Yeast Fermentation Broth (YFB) (peptone 7.5 g/L, yeast extract 4.5 g/L; 1 ml of 1.6% (w/v) bromothymol blue as an indicator) with autoclaved 6% (w/v) lactose. The Durham tubes were also placed into the media to trap the carbon dioxide released. The changes from green to yellow indicated that yeast using the carbon source, that is, lactose.

#### 2.4.3. Stress Exclusion Test [29]

In this test, the isolates were subsequently grown in different conditions that mimicked the various stresses. Firstly, the isolates were grown onto YPG medium and incubated at 30°C for 3 days. From that, a single colony was transferred and grown on YPG medium and incubated at 37°C for another 3 days. Again, a colony was selected and subcultured to YPG 8% (v/v) ethanol and incubated at 30°C for 3 days. A single isolated colony was further subcultured on YPG supplemented with 20% (w/v) glucose and incubated under the same conditions. Finally, yeast cells were transferred on YP medium supplemented with 2% (w/v) sucrose and 8% (v/v) ethanol and incubated under the same conditions.

#### 2.4.4. Hydrogen Sulfide Test [30]

The yeast isolates were grown on lead acetate medium (40 g/L glucose, 5 g/L yeast extract, 3 g/L peptone, 0.2 g/L ammonium sulphate, 1 g/L lead acetate, and 20 g/L agar) and incubated at 30°C for 7 days.

#### 2.4.5. Flocculation Test

The yeast isolates were inoculated in 10 ml of YPG broth and incubated at 30°C for 3 days. They were agitated to observe the flocculation forming.

#### 2.4.6. Temperature Tolerance Test

Yeast isolates were cultured on YPG agar and incubated at 25°C, 30°C, 37°C, and 45°C for 72 h. Growth was observed and analyzed [29].

#### 2.4.7. Carbohydrate Utilization Test [28]

The carbohydrate utilization test was performed using broth (peptone: 10 g; NaCl: 5 g; phenol red: 0.018 g; distilled water: 1000 ml; carbohydrate: 10 g) along with inverted Durham tubes in the broth. The carbohydrates used were dextrose, fructose, lactose, galactose, maltose, and sucrose. The media were inoculated with yeast strains and incubated for 24 hrs. The color change from red to yellow indicated the fermentation using carbon sources.

#### 2.4.8. Hyperosmotic Tolerance Test

Yeast isolates were cultured on YPD broth containing 30, 40, and 50% dextrose and incubated at 30°C for 48 hours. The cell density of different yeast isolates in response to high dextrose concentration was taken.

#### 2.4.9. Ethanol Tolerance Test [29]

Yeast isolates were grown in YPG broth containing 3 different concentrations of ethanol, that is, 10%, 13%, and 15% (v/v), respectively, and incubated at 30°C for 72 hours.

#### 2.4.10. Biomass Comparison in Different Sugars

YPD broth modified with respective sugars was used. Cell density of yeast isolates in sucrose and glucose media was compared. The cell density was measured using DEN-1B Grant bio Densitometer. 18 phi test tubes were used for generating data and data were obtained in McFarland standards.

#### 2.4.11. Invertase Activity Test

*(1) Enzyme Production.* The media for the production of enzyme consist of the following (g/l): sucrose 20, yeast extract 10, ammonium sulphate 1.0, magnesium sulphate 0.75, and potassium dihydrogen phosphate 3.5 with final pH 5. After inoculation with yeast strains, the media were incubated at 30°C in incubator shaker at 120 rpm for 48 hours. After 48 hours, supernatant was harvested by centrifugation at 10000 rpm for 10 minutes at 4°C. This supernatant was used as crude enzyme extract.

*(2) Enzyme Assay [31]*. Slightly modified Sumner and Howells method was used for determining invertase activity. Enzymatic reaction was prepared by incubating 0.1 mL of enzyme solution with 0.9 mL of sucrose in 0.03 M acetate buffer (pH 5.0) for 5 minutes at 30°C. Reaction was stopped with addition of 1 ml of dinitrosalicylic acid reagent and was heated in boiling water bath for 15 minutes. Finally, absorbance was taken at 540 nm in UV-1800 Shimadzu Spectrophotometer.

#### 2.4.12. Cultivation and Dough Leavening

Cultivation of the yeast strains was done in conical flasks in 250 ml media containing yeast extract, peptone, and sucrose. The flasks were kept in shaker incubator at 30°C for 72 hours and yeast pellets were collected after centrifugation at 10,000.

For each strain, 50-gram wheat flour was weighed and 1% salt was mixed with the flour. 6% sugar was dissolved in lukewarm water and 0.6 g yeast pellets were inoculated in the sugar solution to allow its activation. The activated yeast solution was poured in the flour and mixed well. The DY5 which is a commercial yeast strain was used as a positive control, whereas the dough without any yeast was used as a negative control. Proofing was done by incubating the dough at 30°C for 2 hours. It was then baked in hot air oven at 180°C for 20 minutes.

Besides, to assess the rise of dough level, about 10 g of dough mixture was kept in measuring cylinder. It was incubated and the level was noted every half hour.

## 3. Results and Discussion

The colonies exhibiting characteristics such as creamy to white color, fluffy, and smooth margin were selected as tentative* Saccharomyces *strain. As reported by Graeme and Nia, in 2005, cream colonies are the characteristic of yeast especially* Saccharomyces* strain.

The isolation tests as described previously were used for identification of* S.* strain. Out of 26 selected tentative yeast colonies, 14 showed negative lactose utilization result; that is, they were unable to utilize lactose and thus were possible* Saccharomyces *strains.* S. cerevisiae* can be identified due to its ability to ferment sucrose, maltose, fructose, glucose, galactose, and raffinose but not lactose (Thais et al., 2006) as the strain lacks lactase or *β*-galactosidase system [32]. In addition, 17 strains gave negative result in nitrate utilization test which means they were unable to utilize nitrate as sole source of carbon and could be considered as a possible* Saccharomyces* strain. The yeasts of the genera* Saccharomyces* and* Schizosaccharomyces* are unable to use nitrate or nitrite as sole nitrogen source [33]. From these two tests, 14 isolates were selected. The isolate DY5 was obtained from dry yeast sample from the local market to be used as a reference.

**Figure 1:**
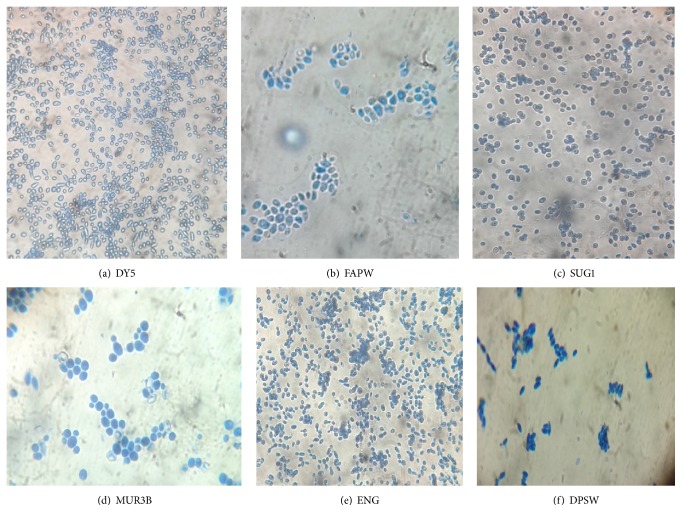
Microscopic observation of yeast colonies from different sources as observed at 100x magnification.

*S. cerevisiae* has a characteristic ellipsoid or ovoid shape [34]. AT 100x, the strains, which showed morphology similar to commercial yeast DY5, were oval in shape, and showed high budding rate, were selected (Figure 1). The strains ENG and MUR3B showed highest budding rate which indicates the higher growth rate and active fermentation as described previously [35].

Therefore, the screening of* Saccharomyces *spp. from the 30 isolates was done by studying the colony characteristics, cell morphology, and lactose utilization pattern and nitrate reduction test. Eight isolates were selected as final candidates for further biochemical tests. The final screened isolates were from Murcha (MUR3B), grape (ENG), Dar (DPSW), sugarcane (SUG1 and SUGW), apple (FAPW), jackfruit (JAK3), and the commercial strain (DY5).

All strains except JAK3 and FAPW showed flocculation property (Table 1). The strains exhibiting good flocculating ability are commercially beneficial as they are easier to separate from media without additional filtration and centrifugation steps during bulk production at industrial scale [36]. Yeast strains producing high levels of hydrogen sulfide are undesirable because H_2_S imparts bad odor and flavor compromising the quality of the bread [37]. All the other strains were acceptable as baker's yeast as the standard commercial strain DY5 produced the highest amount of H_2_S comparatively.

The viability of isolated strains was checked under spontaneous stress conditions such as temperature 37°C, ethanol 8%, sucrose 2%, and glucose 20% for 15 days. It is necessary to check the capacity of baker's yeast to survive these stress conditions as they mimic the ethanol, osmotic, and temperature stress that yeast faces during baking. In the presence of these stresses, yeast becomes impaired and its survival denotes that yeast can carry out fermentation even in these harsh conditions [38, 39]. Also, the last step of the test that checks the tolerance to sucrose may be an important indication of high invertase activity [40]. In this study, every isolate except JAK3 could withstand the series of stress and was capable of adapting to the extreme conditions (Table 2). These isolates, thus, could be potential leavening agent in bread making.

In response to temperature change, all the strains including commercial yeast strain were able to grow at temperatures 25°C, 30°C, and 37°C. Strains JAK3 and SUGW showed feeble growth at 37°C. However, the strain SUG1 was able to survive at higher temperature (45°C) too at which even the commercial strain failed to grow (Table 3). It indicated that they could have better potential in bread making to speed up the proofing process, with increased carbon dioxide production [41].

In sugar utilization test, change of color to yellow signifies that the organisms utilize sugar to produce acidic products and gas which is trapped in Durham tubes. The orange color indicates the partial fermentation of sugars. All the isolates were able to ferment sugars to different extent (Table 4). However, the three strains MUR3B, ENG, SUG1, and DPSW showed sugar utilization capability comparable to the commercial strain, that is, DY5. MUR3B showed maximum gas production in all the sugars. This strain produced maximum gas in fructose compared to DY5. Since gas production is the most important criteria for leavening and can indicate the high invertase activity, strains can be selected on the basis of gas production too [36].

**Figure 2:**
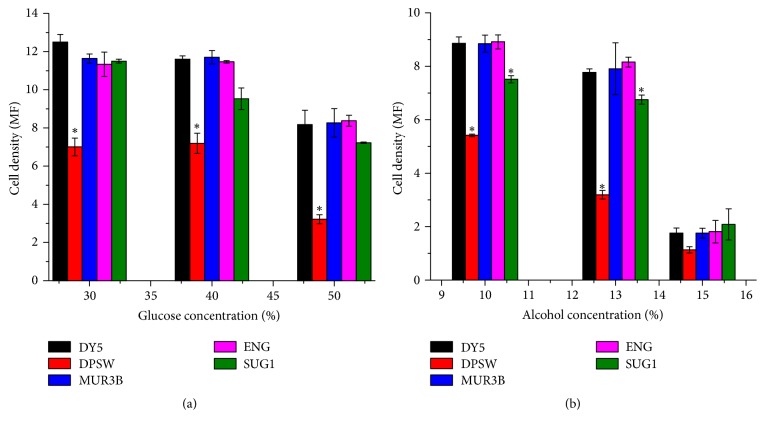
Comparison of osmotolerance (a) and alcohol tolerance (b) of different yeast isolates with commercial yeast. ^*∗*^The mean difference is significant at 0.02 level when comparing means between DY5 and other strains.

In baking process, it is very important for a yeast strain to survive the minimal osmotic pressure [22]. Osmotic pressure of the medium is vital in determining the cell viability [24]. During osmotic stress, cell dies due to several reasons such as water efflux across the membrane [42], removal of water molecules causing structural modifications of phospholipids [43], osmotic mass transfer [44], and toxicity caused by high solutes concentration [45]. The one-way between-groups analysis was performed to observe the effect of high glucose concentration which corresponds to osmotic pressure on growth of different yeast species, as measured by cell density. Yeast strains were cultured at three different glucose concentrations (30%, 40%, and 50%) and the growth of the strains at these concentrations was compared to that with commercial strains DY5. There was a statistically significant difference at the *P* < 0.02 level in cell density for the three glucose concentrations: 30%, 40%, and 50% glucose concentration (Figure 2(a))

The effective size, calculated using eta squared, was 0.917 for 30%, 0.909 for 40%, and 0.883 for 50% glucose concentration. Post hoc comparisons using Tukey's HSD test indicated that the mean cell density for DPSW in both 30% and 40% glucose concentration was significantly lower than that of DY5. The mean cell density of other strains did not differ significantly compared to that of commercial dry yeast (DY5) in the three given glucose concentrations. There was significant decrease to the biomass of all strains in 50% glucose concentration with respect to the biomass of respective strains at 40%. In 30% and 40%, glucose concentration MUR3B and ENG shows the same level of tolerance to that of DY5. This means they can tolerate a higher osmotic pressure than the other strains including commercial yeasts.

The one-way between-groups analysis was performed to observe the alcohol tolerance of different yeast strains, as measured by cell density in presence of different alcohol concentrations (Figure 2(b)). It is necessary to check the tolerability and viability of yeast strains at different alcohol concentrations because alcohol is produced as a secondary metabolite that increases the flavor of bread during leavening [46]. Yeast strains were cultured at three different alcohol concentrations (10%, 13%, and 15%). At 10% alcohol concentration, the strains MUR3B and ENG showed tolerance to alcohol that was comparable to DY5. The strain ENG that was isolated from grapes showed a better cell growth than commercial yeast, that is, DY5. However, the post hoc comparisons indicated that the mean cell density for DPSW and SUG1 in 10% alcohol concentration was significantly lower than that of DY5. Similarly, at 13% alcohol concentration, the strain MUR3B and ENG showed better tolerance to alcohol compared to DY5 but the mean cell density for DPSW and SUG1 was significantly lower than that of DY5 at 13% alcohol concentration too. ENG and MUR3B showed ethanol tolerance that is comparable to DY5. Some level of growth was seen at 15% alcohol concentration also but the biomass production was significantly lower than that of 13%. So these strains showed some level of alcohol tolerance at 15% and are tolerant to 13% [47]. Ethanol damages the mitochondrial DNA and causes the inactivation of enzymes such as hexokinases and dehydrogenase and if the alcohol concentration is too high, it destroys the cell membranes of yeast and kills them by impairing their growth [48].

**Figure 3:**
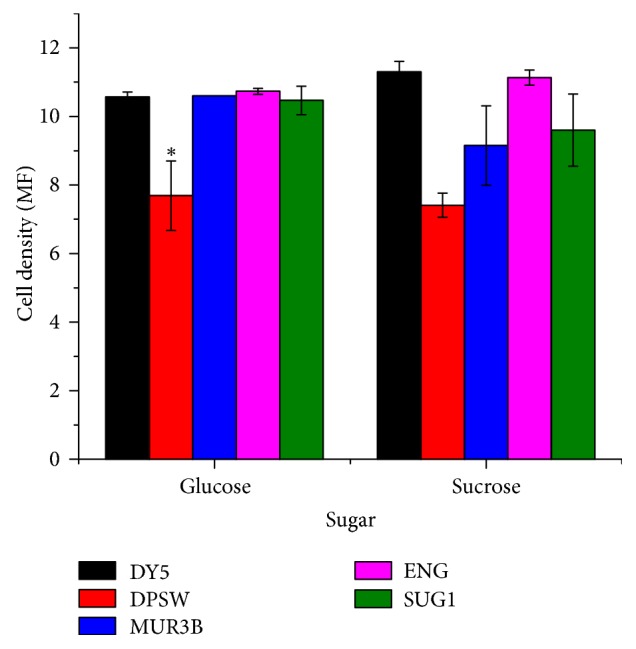
Comparison of different yeast isolates with commercial yeast in presence of sugar. Cell density comparison of different yeast isolates with commercial yeast in presence of glucose and sucrose. ^*∗*^The mean difference is significant at 0.02 level when comparing means between DY5 and other strains.

The growth of different strains was checked in glucose and sucrose by measuring the cell density (Figure 3). In glucose, the cell density of MUR3B, ENG, and SUG1 was very similar to the cell density of commercial strain DY5. However, the growth of DPSW was very low compared to DY5. In sucrose, only the cell density of ENG was very similar to DY5. Other strains showed less growth compared to commercial strain.

**Figure 4:**
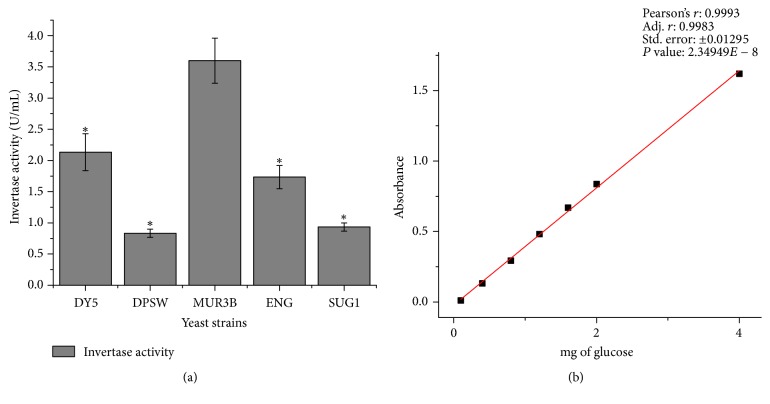
(a) Invertase activity of different yeast strains; (b) standard curve of mg of glucose versus absorbance. ^*∗*^The mean difference is significant at 0.02 level when comparing means between MUR3B and other strains.

The invertase activity for the strain MUR3B was found to be the highest (Figure 4(a)). The activity of the other strains including commercial yeast was significantly lower than that of MUR3B. The strains having high level of invertase activities have found to be less capable of overcoming osmotic pressure [22] but the MUR3B strain that exhibits highest level of invertase activity also shows growth comparable to commercial strain at highest concentration of glucose in osmotic test. One unit of invertase is defined as amount of enzyme which liberates one mole of glucose/minute/mL under the assay condition (optimal pH (4.8), temperature (40°C), and initial sucrose concentrations of 5 mM).

## 4. Dough Fermentation and Baking

**Figure 5:**
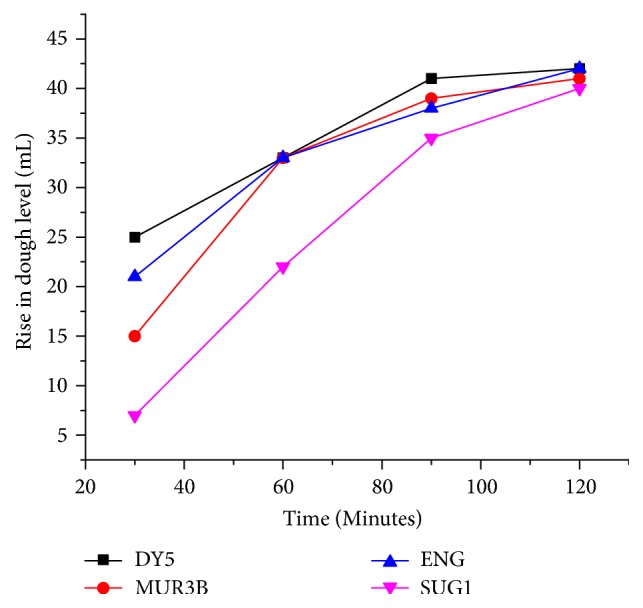
Comparison of leavening effect of different yeast strains.

The rise of dough level or the speed of fermentation process was assessed by noting the rise of dough mixture kept in measuring cylinder (Figure 5). Although the rise in DY5 was the fastest, the strains ENG and MUR3B showed similar rise. Since the strain DPSW showed poor performance in ethanol tolerability, cell density, and hyperosmotic tolerance, it was not used in dough making process.

**Figure 6:**
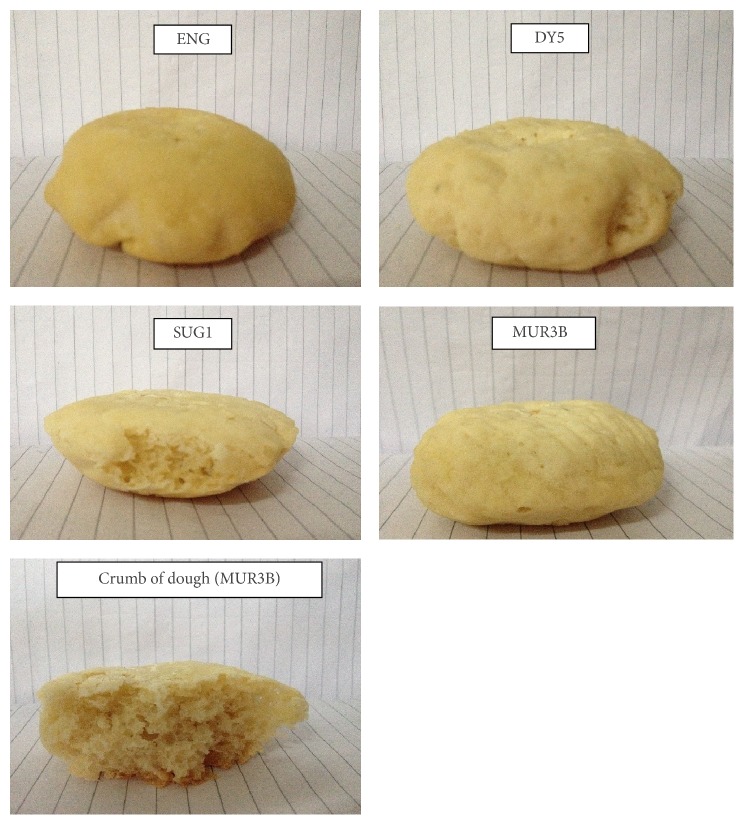
Baked dough from different isolated strains: SUG1, MUR3B, ENG, and DY5.

The final fermented dough with SUG1 and ENG was comparable to that by the commercial strain DY5. The cross-sectional examination of the dough from all strains also showed similar crumb textural property (Figure 6). The properties of the baked dough from the three strains SUG1, MUR3B, and ENG were found similar to the dough fermented by the commercial yeast strain (DY5) (Table 5).

## 5. Conclusion

This study showed that three isolated strains of* S. cerevisiae *from native sources could be used as a commercial baker's yeast. The three strains, all being* Saccharomyces cerevisiae, *are MUR3B from Murcha, ENG from grapes, and SUG1 from sugarcane; they show strong potential for commercialization after appropriate research on biomass optimization culture preservation. These strains can give unique flavor to bread and can form an identity for the bread industry of Nepal. Our study finds that the indigenous plant “Dar” inhabits yeast, but this yeast strain could not be used as baker's yeast because of its poor leavening properties. Meanwhile, the strains from Murcha, grapes, and sugarcane are potential sources for baker's yeast.

## Figures and Tables

**Table 1:** Flocculation test.

S. number	Samples	Flocculation	Hydrogen sulfide production
(1)	MUR3B	Yes	++
(2)	ENG	Yes	+
(3)	DPSW	Yes	−−
(4)	FAPW	No	++
(5)	DY5 (SC)	Yes	+++
(6)	SUG1	Yes	+
(7)	JAK3	No	+++
(8)	SUGW	Yes	+

+++: intensive response; ++: moderate response; +: low response; −−: no response.

**Table 2:** Stress exclusion tests on yeast cell for temperature and cell osmotic pressure in high concentration of ethanol and sugar.

Samples	Growth into different media					Use of yeast as potential leavening agent
	YPG	Temperature 37°C	Ethanol 8% (v/v)	YPG 20% (v/v) glucose	YPS 2% (v/v) sucrose + ethanol 8% (v/v)	
MUR3B	+++	+++	+++	+++	++	Yes
ENG	+++	++	+++	+++	++	Yes
DPSW	+++	+++	+++	++	++	Yes
FAPW	+++	+++	+++	+++	++	Yes
DY5 (SC)	+++	+++	+++	+++	++	Yes
SUG1	+++	+++	+++	+++	++	Yes
JAK3	+++	++	+++	−−	−−	No
SUGW	+++	+++	++	++	++	Yes

+++: intensive response; ++: moderate response; −−: no response; YPG: yeast peptone glucose medium; YPS: yeast peptone sucrose medium.

**Table 3:** Growth and inhibition of yeast isolates at different growth temperatures.

S. number	Samples	Temperature			
		25°C	30°C	37°C	45°C
(1)	MUR3B	+++	+++	+++	−−
(2)	ENG	+++	+++	+++	−−
(3)	DPSW	+++	+++	++	−−
(4)	FAPW	+++	+++	+++	−−
(5)	DY5 (SC)	+++	+++	+++	−−
(6)	SUG1	+++	+++	+++	++
(7)	JAK3	+++	++	++	−−
(8)	SUGW	+++	+++	++	−−

+++: intensive response; ++: moderate response; −−: no response.

**Table 4:** Sugar utilization test.

Yeast strains	Dextrose		Fructose		Galactose		Maltose		Sucrose	
	Color	Gas	Color	Gas	Color	Gas	Color	Gas	Color	Gas
DY5	Yellow	+++	Yellow	++	Yellow	++	Yellow	+++	Yellow	+++
DPSW	Yellow	+++	Orange	++	Yellow	++	Yellow	++	Yellow	+++
MUR3B	Yellow	+++	Yellow	+++	Yellow	+++	Yellow	+++	Yellow	+++
ENG	Yellow	++	Yellow	++	Yellow	+	Orange	++	Yellow	+++
SUG1	Yellow	+++	Yellow	+++	Yellow	++	Yellow	+++	Yellow	+++
FAPW	Orange	+	Orange	−	Orange	++	Orange	++	Yellow	−
JAK3	Orange	−	Orange	+	Yellow	+++	Yellow	+	Orange	+
SUGW	Yellow	++	Orange	+	Orange	−	Yellow	+++	Yellow	++

+: ≤one-third of Durham tube; ++: greater than one-third and ≤two-thirds of Durham tube; +++: greater than two-thirds; −: no gas production.

**Table 5:** Properties of baked dough.

S. number	Sample	Color of crust	Color of crumb	Aroma
(1)	MUR3B	Light Brown	Creamy	+++
(2)	ENG	Light Brown	Creamy	++
(3)	DY5	Light Brown	Creamy	+++
(4)	SUG1	Light Brown	Creamy	+++

+++: baking aroma of commercial active dry yeast; ++: intensity of aroma being less than that of commercial active dry yeast.
